# Corneal lymphangiogenesis ameliorates corneal inflammation and edema in late stage of bacterial keratitis

**DOI:** 10.1038/s41598-019-39876-x

**Published:** 2019-02-27

**Authors:** Akitomo Narimatsu, Takaaki Hattori, Naohito Koike, Kazuki Tajima, Hayate Nakagawa, Naoyuki Yamakawa, Yoshihiko Usui, Shigeto Kumakura, Tetsuya Matsumoto, Hiroshi Goto

**Affiliations:** 1 Department of Ophthalmology, Tokyo Medical University, Tokyo, Japan; 2 Department of Microbiology, Tokyo Medical University, Tokyo, Japan; 3 Department of Small Animal Internal Medicine, School of Veterinary Medicine, University of Kitasato, Aomori, Japan; 4 Department of Infectious Diseases, International University of Health and Welfare, Narita, Japan

## Abstract

Lymphatic vessels play a crucial role in systemic immune response and regulation of tissue fluid homeostasis. Corneal lymphangiogenesis in bacterial keratitis has not been studied. In this study, we investigated the mechanism and the role of corneal lymphangiogenesis in a murine bacterial keratitis model using *Pseudomonas aeruginosa*. We first demonstrated that corneal lymphangiogenesis was enhanced mainly in the late stage of bacterial keratitis, contrary to corneal angiogenesis that started earlier. Corresponding to the delayed lymphangiogenesis, expression of the pro-lymphangiogenic factors VEGF-C and VEGFR-3 increased in the late stage of bacterial keratitis. We further found that F4/80 and CD11b positive macrophages played an essential role in corneal lymphangiogenesis. Notably, macrophages were specifically involved in corneal lymphangiogenesis in the late stage of bacterial keratitis. Finally, we demonstrated the beneficial role of corneal lymphangiogenesis in ameliorating the clinical course of bacterial keratitis. Our study showed that bacterial activity was not directly involved in the late stage of keratitis, while corneal lymphangiogenesis reduced corneal edema and clinical manifestation in the late stage of bacterial keratitis. These findings suggest that the process of lymphangiogenesis in bacterial keratitis ameliorates corneal inflammation and edema in the late stage of bacterial keratitis.

## Introduction

The lymphatic vascular system plays an important role in tissue fluid homeostasis and systemic immune response^1^. Previous studies have demonstrated that many factors activated lymphangiogenesis; including fibroblast growth factor^2^, platelet-derived growth factor^3^, and vascular endothelial growth factor (VEGF)-A, -C and -D^4,5^. In particular, VEGF-C signaling through VEGF receptor-3 (VEGFR-3) plays an essential role in embryonic development and postnatal lymphangiogenesis^6,7^. Several studies have reported that VEGF-C‒induced angiogenesis was minimal compared to lymphangiogenesis^4,8^, whereas VEGF-A signaling through VEGFR-2 was more important for angiogenesis than for lymphangiogenesis^9,10^.

The transparency of normal cornea is maintained due to the unique immune environment in the eye that maintains avascularity in corneal tissue^11^. However, previous studies in animals and humans showed that inflammatory damage elicited corneal angiogenesis and lymphangiogenesis^12–14^. Thus, the mouse cornea is appropriate for observation of lymphangiogenesis and angiogenesis. The mechanism by which how lymph vessel spouts into inflamed corneas was well investigated using various models such as suture placement^4,13,14^, alkali burn^15^, thermal injury^16^, herpetic keratitis^17,18^, dry eye^19^, cornea transplantation^20,21^ allergic disease^22^ and acute corneal edema^23^. Based on these studies, corneal lymphangiogenesis is mainly promoted by VEGF-C through VEGFR-3 signaling. On the other hand, several studies have focused on the mechanism of corneal lymphangiogenesis induced by macrophages. Macrophages are known to promote corneal lymphangiogenesis via the VEGF-C‒VEGFR-3 pathway^4,24^. Furthermore, it is reported that CD11b positive macrophages directly transform to lymphatic endothelial cells and form tube structures, suggesting a distinctive mechanism of corneal lymphangiogenesis^13^.

*Pseudomonas aeruginosa* is a representative Gram-negative bacterial strain that causes contact lens–related bacterial keratitis. Antibiotics are the major treatment of bacterial keratitis, but residual corneal opacification may cause visual disturbance in patients^25,26^. Lipopolysaccharide (LPS) is essential for viability of *Pseudomonas aeruginosa*^27^, and immune recognition of LPS through the Toll-like receptor (TLR)4 pathway leads to the production of pro-inflammatory cytokines such as IL-6 and tumor necrosis factor-α^28^. In addition, Previous study of extraocular tissues has reported that LPS also activates VEGF-C via the TLR4 pathway and subsequently induces lymphangiogenesis^29^. Moreover, TLR4 in lymphatic endothelial cells play an important role in LPS-induced lymphangiogenesis by recruitment of macrophages in mice diaphragm^30^. In corneas, macrophages have been shown to control inflammatory response caused by neutrophils, bacterial activity and inflammatory cytokines in *Pseudomonas aeruginosa*-induced keratitis^31,32^. However, whether bacterial inoculation causes corneal lymphangiogenesis has not been studied.

Regarding the role of corneal lymphatic vessel, several studies have shown both beneficial and undesirable effects of corneal lymphangiogenesis. Several studies have reported that corneal lymphangiogenesis deteriorates herpetic keratitis^18^, dry eye^19^, allergic disease^22^, and corneal transplant survival^20^, suggesting the adverse effects of corneal lymphangiogenesis. Conversely, recent studies have reported that lymphangiogenesis plays a beneficial role in the regulation of corneal edema in acute corneal edema, suggesting that lymphatic vessels may contribute to reduce immune cells and edema^23,33^. From these reports, the role of corneal lymphangiogenesis remains controversial and lymphangiogenesis may play different roles in different corneal pathological conditions. Furthermore, the role of lymphangiogenesis during bacterial keratitis has not been studied.

In this study, we investigated the mechanism and the role of corneal lymphangiogenesis in a murine bacterial keratitis model using *Pseudomonas aeruginosa*. Our results showed that macrophages were involved in corneal lymphangiogenesis in the late stage of bacterial keratitis. Furthermore, our findings suggest that corneal lymphangiogenesis has an important role in the resolution of bacterial keratitis.

## Results

### Bacterial keratitis led to corneal lymphangiogenesis and angiogenesis

**Figure 1:**
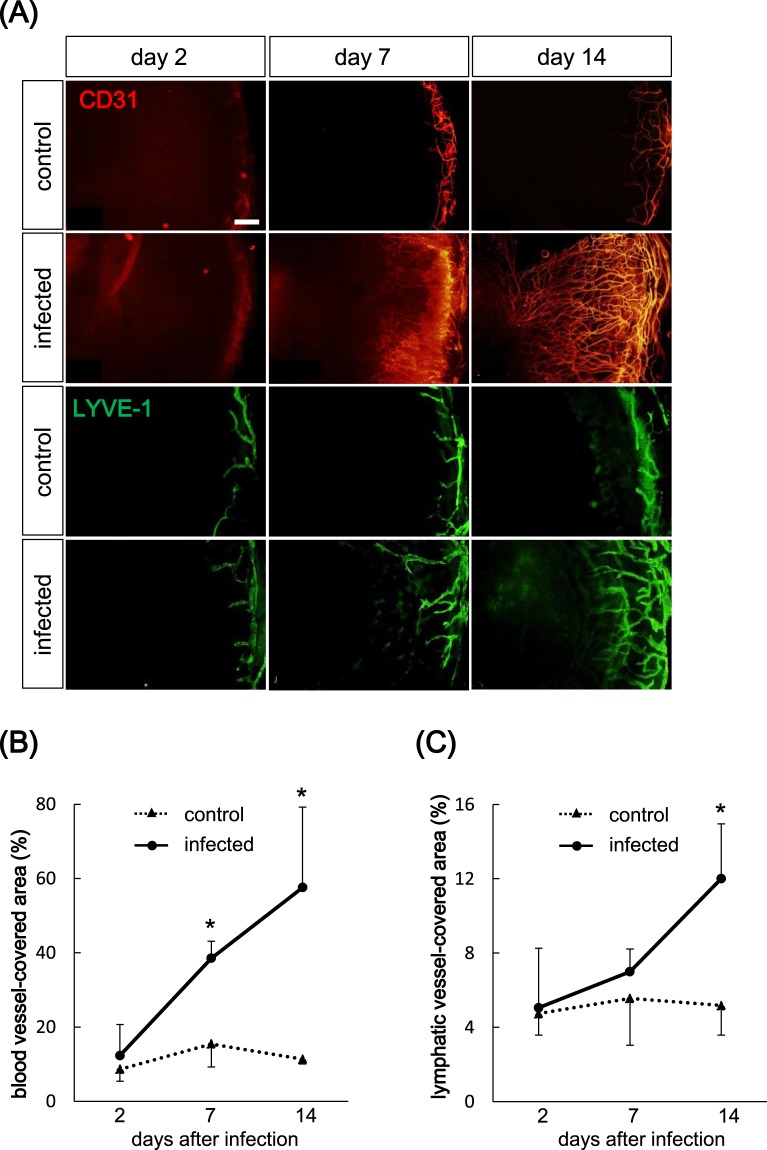
Bacterial keratitis induced corneal angiogenesis and lymphangiogenesis. (**A**) Time courses of corneal angiogenesis and lymphangiogenesis in bacterial keratitis. Whole-mounted corneas were immunostained with anti-CD31 antibody as marker of blood vessels (red) and anti-LYVE-1 antibody as marker of lymphatic vessels (green), and images were captured under a fluorescence microscope on days 2, 7 and 14 post-inoculation. Scale bar: 200 μm. (**B**,**C**) Time courses of percentage of area of blood vessels and lymphatic vessels in bacterial keratitis (●) compared to control (▲) (n = 5/group, *p < 0.05). Data are expressed as mean ± SD (error bar). The results are representative of three experiments.

We first examined whether bacterial keratitis leads to corneal lymphangiogenesis and angiogenesis. For this purpose, we used the established mouse bacterial keratitis model using *Pseudomonas aeruginosa*^34^. *Pseudomonas aeruginosa* strain PAO-1 (2.5 × 10^5^ CFU/2.5 μl) was inoculated onto mouse cornea after the corneal epithelium was scratched using a 27-G needle. Control mice were inoculated with phosphate buffered saline (PBS). Corneas were harvested from these mice on days 2, 7 and 14 post-inoculation. Corneal angiogenesis and lymphangiogenesis were evaluated by immunostaining of anti-CD31 antibody and anti-LYVE-1 antibody, respectively, and examined by fluorescence microscopy. Results showed extension of blood and lymphatic vessels into the cornea in the infected group compared to the control group on days 7 and 14 post-inoculation (Fig. 1A). When the areas of blood and lymphatic vessels (percentage) were calculated in the two groups, angiogenesis increased significantly on days 7 (infected: 38.52% versus control: 15.44%, p < 0.05) and 14 (infected: 57.57% versus control: 11.34%, p < 0.05) post-inoculation in the infected group compared to controls (Fig. 1B). On the other hand, the percentage of lymphatic vessels increased significantly in the infected group on day 14 post-inoculation (infected: 12.00% versus control: 5.17%, p < 0.05; Fig. 1C), although there was no significant difference between the infected and control groups on day 7 post-inoculation (infected: 6.99% versus control: 5.55%, p = 0.34; Fig. 1C). These data indicated that lymphangiogenesis was involved in bacterial keratitis, and that corneal lymphangiogenesis was mainly enhanced in the late stage of bacterial keratitis as compared to earlier increase of corneal angiogenesis.

### Increased expression of lymphangiogenic factors VEGF-C and VEGFR-3 in late stage of bacterial keratitis

**Figure 2:**
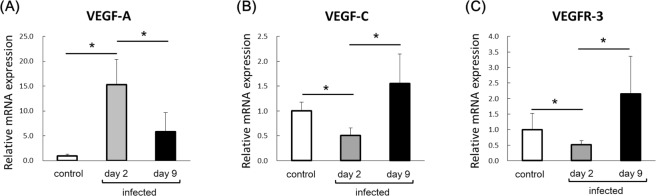
Time courses of VEGF-A, VEGF-C and VEGFR-3 mRNA expressions in bacterial keratitis. Expression levels of these factors in corneas were analyzed by quantitative real-time PCR. Corneas inoculated with PBS after scratching on day 2 post-inoculation were used as controls. (n = 10/group, *p < 0.05). Data are expressed as mean ± SD (error bar). The results are representative of two experiments.

VEGF-A is known to be a primary factor of angiogenesis^9,10^. On the other hand, VEGF-C and VEGFR-3 have an important role in the development of lymphatic vessels^6,7^. By immunostaining blood vessels and lymphatic vessels, our findings showed that corneal lymphangiogenesis was especially increased in the late stage of bacterial keratitis (Fig. 1). We next investigated the time courses of VEGF-A, VEGF-C and VEGFR-3 expression in bacterial keratitis. We harvested corneas from mice with bacterial keratitis on days 2 and 9 post-inoculation as the respective starting time of angiogenesis and lymphangiogenesis. Messenger RNA expressions of VEGF-A, VEGF-C and VEGFR-3 was examined by real-time RT-PCR. Corneas from mice scratched and inoculated with PBS on day 2 post-inoculation were used as controls. VEGF-A mRNA expression was significantly upregulated on days 2 and 9 post-inoculation compared to control. Moreover, VEGF-A expression showed significant downregulation on day 9 compared to day 2 post-inoculation (Fig. 2A). Interestingly, mRNA expression of the lymphangiogenic factors VEGF-C and VEGFR-3 were significantly downregulated compared to controls on day 2 post-inoculation. On the other hand, both VEGF-C and VEGFR-3 mRNA expressions were significantly upregulated compared to control on day 9 post-inoculation (Fig. 2B,C). These results confirmed that corneal lymphangiogenesis was specifically activated in the late stage of bacterial keratitis.

### Macrophage depletion in the late stage of bacterial keratitis specifically inhibited corneal lymphangiogenesis

Several studies have reported that activated macrophages promote corneal lymphangiogenesis^4,13^. Thus, we first confirmed the localization of macrophages and corneal lymphatic vessels in the late stage of bacterial keratitis under confocal microscopy, by immunostaining using anti-LYVE-1 antibody, anti-CD11b antibody and anti-F4/80 antibody (Supplementary Fig. S1). Both CD11b positive and F4/80 positive macrophages were increased in infected corneas compared to non-infected corneas. Furthermore, in infected corneas, co-localization of CD11b positive or F4/80 positive macrophages with corneal lymphatic vessels was observed on day 14 post-inoculation. We further investigated whether depletion of macrophages using clodronate liposomes reduces corneal lymphangiogenesis in bacterial keratitis (Supplementary Fig. S2).

In clodronate (+) group, the clodronate liposomes were injected at the same time as bacterial inoculation. Injection of clodronate liposomes resulted in reduction of both CD11b positive macrophages and F4/80 positive macrophages in the infected corneas on day 2 post-inoculation, indicating successful macrophage depletion. However, re-infiltration of both CD11b positive macrophages and F4/80 positive macrophages was observed on day 5 post-inoculation.

**Figure 3:**
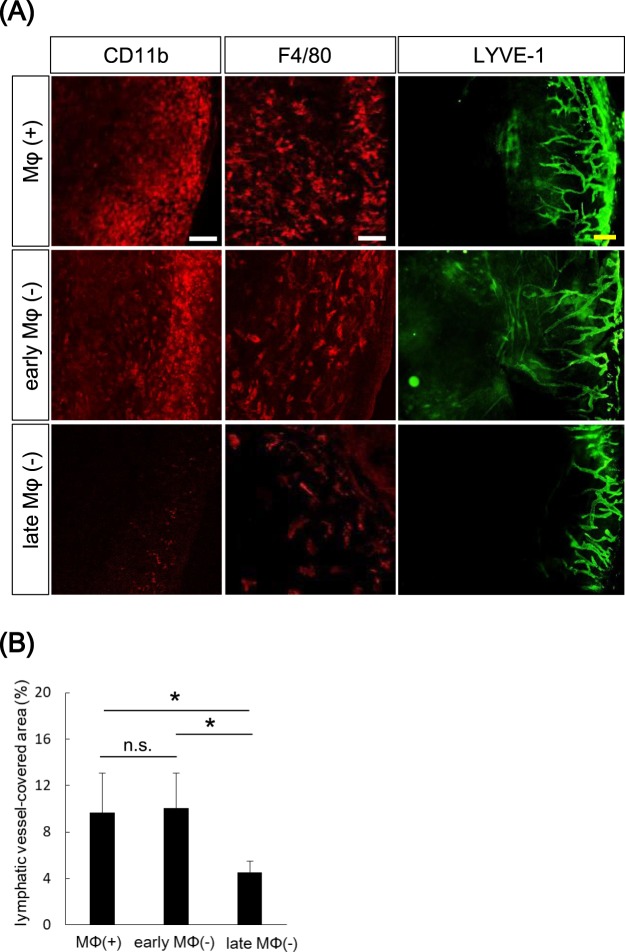
Macrophage depletion in the late stage of bacterial keratitis specifically inhibited corneal lymphangiogenesis. (**A**) Lymphangiogenesis and infiltration of macrophages in the cornea were compared between mice with macrophages depleted in early stage [early macrophage (−) group] and those with macrophages depleted in late stage of bacterial keratitis [late macrophage (−) group]. In early macrophage (−) group, clodronate liposomes were injected on days −2 (day 2 pre-inoculation), 2, and 6 post-inoculation. In late macrophage (−) group, the clodronate liposomes were injected on days 4, 8 and 12 post-inoculation. Control mice were not injected clodronate liposomes [macrophage (+) group] Corneas were immunostained by anti-CD11b antibody, anti-F4/80 antibody as markers of macrophages (red) and anti-LYVE-1 antibody as marker of lymphatic vessel (green) on day 14 post-inoculation. Scale bar: 100 μm (white), 200 μm (yellow). (**B**) Lymphatic vessel-covered areas were compared among three groups (n = 5/group, *p < 0.05; n.s.: not significant). MΦ: macrophage. Data are expressed as mean ± SD (error bar). The results are representative of two experiments.

We next evaluated lymphangiogenesis by depletion of macrophages in corneas with bacterial keratitis. We examined lymphangiogenesis and infiltration of macrophages in the cornea in different stages of bacterial keratitis by changing the time of intraperitoneal clodronate liposome injection. To deplete macrophages in the early stage of keratitis, clodronate liposomes were injected on days −2 (day 2 pre-inoculation), 2, and 6 post-inoculation [early macrophage (−) group]. To deplete macrophages in the late stage of keratitis, clodronate liposomes were injected on days 4, 8 and 12 post-inoculation [late macrophage (−) group]. Control mice did not receive clodronate liposome injection [macrophage (+) group]. Corneas were harvested from each group, and stained with anti-LYVE-1 antibody, and percentage of lymphatic vessel area was measured. In the early macrophage (−) group, both CD11b macrophages and F4/80 positive macrophages were not reduced on day 14 post-inoculation. In contrast, in the late macrophage (−) group, both CD11b positive macrophages and F4/80 positive macrophages were greatly reduced (Fig. 3A). Lymphangiogenesis was not reduced in the early macrophage (−) group compared to control (early macrophage (−): 10.03% versus control: 9.65%, p = 0.85; Fig. 3A,B), while significant reduction in lymphangiogenesis was observed in the late macrophage (−) group compared to control (late macrophage (−): 4.54% versus control: 9.65%, p < 0.05; Fig. 3A,B). Hence, these data indicated that macrophages were involved in corneal lymphangiogenesis in the late stage of bacterial keratitis. Moreover, corneal lymphangiogenesis was inhibited by depleting macrophages in the later stage of bacterial keratitis.

### Corneal lymphangiogenesis in bacterial keratitis contributed to amelioration of corneal opacity and edema

It remains controversial whether corneal lymphangiogenesis plays a beneficial or undesirable role in the clinical and pathological conditions of cornea^18–21,23,33^. Therefore, we next examined the role of corneal lymphangiogenesis in bacterial keratitis.

**Figure 4:**
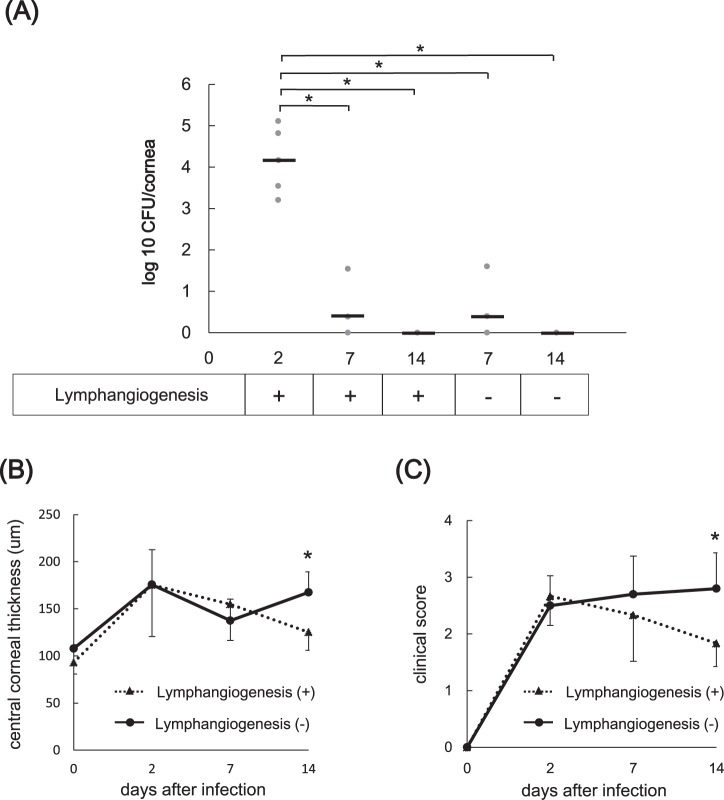
Comparisons of bacterial count in cornea, central corneal thickness, and clinical score in lymphangiogenesis (−) and lymphangiogenesis (+) groups. (**A**) The time courses of bacterial load in corneas inoculated with bacteria in mice with and without lymphangiogenesis were obtained using CFU assay. In lymphangiogenesis (−) group, corneal lymphangiogenesis was inhibited by intraperitoneal injection of clodronate liposome on days 4, 8 and 12 post-inoculation (n = 5/group, *p < 0.05). Circles represent the data obtained from individual corneas; horizontal bars represent mean data in each group. (**B**) Comparison of central corneal thickness measured by AS-OCT as a measure of corneal edema in lymphangiogenesis (+) group (▲) versus lymphangiogenesis (−) group (●) (n = 5/group, *p < 0.05). (**C**) Comparison of clinical infection score in lymphangiogenesis (+) group (▲) versus lymphangiogenesis (−) group (●) (n = 5/group, *p < 0.05). For B and C, data are expressed as mean ± SD (error bar). The results are representative of two experiments.

First, to determine the involvement of bacterial activity in the late stage of bacterial keratitis, we evaluated the time course of bacterial load using CFU assay. The corneas were harvested and homogenized on days 2, 7 and 14 post-inoculation. The samples were plated on *Pseudomonas aeruginosa* isolation agar, and the numbers of colonies on the plates were counted. As shown in Fig. 4A, the bacterial count in the cornea decreased significantly on days 7 and 14 compared to day 2 post-inoculation. Notably, bacteria were not detected on day 14 post-inoculation. Subsequently, we sought to confirm whether the reduction of corneal lymphangiogenesis induced by macrophage depletion was associated with the bacterial count. In lymphangiogenesis (−) group, corneal lymphangiogenesis was inhibited by intraperitoneal injection of clodronate liposomes on days 4, 8 and 12 post-inoculation. The bacterial counts were the same regardless of the presence or absence of lymphatic vessels on days 7 and 14 post-inoculation (Fig. 4A). These results indicated that bacterial activity was not involved in the late stage of bacterial keratitis regardless of the presence or absence of lymphatic vessels.

We further evaluated whether corneal lymphangiogenesis in bacterial keratitis was associated with corneal edema. Corneal edema was evaluated by central corneal thickness (CCT) using anterior segment optical coherence tomography (AS-OCT), as described previously^20^. Corneal lymphangiogenesis was inhibited by intraperitoneal injection of clodronate liposomes on days 4, 8 and 12 post-inoculation [lymphangiogenesis (−) group]. In lymphangiogenesis (+) group, the maximum CCT was reached on day 2 post-inoculation (175.4 μm), followed by a gradual decline on days 7 to 14 post-inoculation (day 7: 154.8 μm versus day 14: 125.2 μm, p < 0.05).

In the lymphangiogenesis (−) group, CCT also reached the maximum on day 2 post-inoculation (175.6 μm). However, CCT on day 14 was significantly increased compared to day 7 post-inoculation (day 7: 137.5 μm versus day 14: 167.5 μm, p < 0.05; Fig. 4B, Supplementary Fig. S3A). In addition, CCT in the lymphangiogenesis (−) group was significantly greater compared to that in the lymphangiogenesis (+) group on day 14 post-inoculation [lymphangiogenesis (−): 167.5 μm versus lymphangiogenesis (+): 125.2 μm, p < 0.05]. These results indicated that corneal lymphangiogenesis in bacterial keratitis was associated with corneal edema.

We finally investigated the effect of corneal lymphangiogenesis on the clinical course of bacterial keratitis. We evaluated clinical infection score in both the lymphangiogenesis (+) and lymphangiogenesis (−) groups on days 2, 7 and 14 post-inoculation. In the lymphangiogenesis (−) group, lymphangiogenesis was inhibited by intraperitoneal injection of clodronate liposomes on days 4, 8 and 12 post-inoculation. As shown in Supplementary Fig. S3B, corneal opacification was observed on day 2 in both lymphangiogenesis (+) and lymphangiogenesis (−) groups. In lymphangiogenesis (+) group, this opacification improved gradually on days 7 and 14 post-inoculation. In contrast, in lymphangiogenesis (−) group, corneal opacification persisted on days 7 and 14 post-inoculation. In the lymphangiogenesis (+) group, clinical infection score reached the maximum on day 2 post-inoculation, and then gradually improved on days 7 and 14 post-inoculation (Fig. 4C). In the lymphangiogenesis (−) group, clinical infection score also rose sharply on day 2 post-inoculation, but plateaued thereafter, showing no remarkable difference between days 7 and 14.The score on day 14 was significantly higher in the lymphangiogenesis (−) group compared to the lymphangiogenesis (+) group. These data showed a similar tendency to those of corneal CCT, suggesting that corneal lymphangiogenesis prevented deterioration of clinical condition in the late stage of bacterial keratitis. From the above results, it may be concluded that corneal lymphangiogenesis contributes to amelioration of corneal opacity resulting from edema and immune response.

## Discussion

In the current study, we revealed the distinctive mechanism of corneal lymphangiogenesis in a murine bacterial keratitis model using *Pseudomonas aeruginosa*, which is the common bacterial species causing contact lens-related bacterial keratitis^25,26^. We further showed that corneal lymphangiogenesis played an important role in the resolution of bacterial keratitis. To our knowledge, this is the first study that elucidates the characteristics of corneal lymphangiogenesis using an *in vivo* bacterial keratitis model.

We first demonstrated that bacterial keratitis led to corneal lymphangiogenesis, which is consistent with the findings obtained from various other corneal injury models^4,13–23^. Our study provides the first direct evidence that corneal lymphatic vessels sprout in bacterial keratitis. Especially, we found that lymphangiogenesis is enhanced mainly in the late stage of bacterial keratitis, contrary to angiogenesis that occurs earlier. Nakao *et al*.^14^ previously reported that corneal angiogenesis was induced by lower concentration of VEGF-A compared to lymphangiogenesis, and angiogenic vessels impeded lymphatic growth by trapping VEGF-C. On the other hand, Wuest *et al*.^17^ reported no delay in corneal lymphangiogenesis compared to angiogenesis in herpetic stromal keratitis. Interestingly, lymphangiogenesis in herpetic stromal keratitis is strictly dependent on VEGF-A signaling through VEGFR-2, and not VEGF-C signaling via VEGFR-3. In our study, VEGF-C and VEGFR-3 gene expressions were significantly upregulated in the late stage compared to the early stage of bacterial keratitis, whereas VEGF-A expression peaked in the early stage and declined in the late stage of bacterial keratitis. Thus, our data suggest that VEGF-C signaling through VEGFR-3 pathway plays a principal role in lymphatic growth in bacterial keratitis.

VEGF-C signaling through the VEGFR-3 pathway is also known to be activated by macrophages^4,24^. Our study showed that CD11b positive and F4/80 positive macrophages were involved in corneal lymphangiogenesis in bacterial keratitis. Furthermore, we found that macrophages specifically enhanced corneal lymphangiogenesis in the late stage of bacterial keratitis, whereas macrophages had no effect on corneal lymphangiogenesis in the early stage of bacterial keratitis. In chronic diseases affecting extraocular tissues, macrophages play crucial roles in both inflammatory and anti-inflammatory mechanisms^35^. It is reported that F4/80 positive macrophages activate the TLR4 pathway and subsequently promote neutrophil infiltrations and bacterial killing^34^. On the other hand, a previous *in vitro* study showed that macrophages switched from pro- to anti-inflammatory role through activated TLR4 signaling depending on the time course of inflammation^36^. Macrophages are also known to recognize LPS via TLR4 signaling in Gram-negative bacteria such as *Pseudomonas aeruginosa*^30^. Thus, there is a high possibility that macrophages also switched to an anti-inflammatory phenotype in the late stage of bacterial keratitis. Hos *et al*.^33^ showed that corneal lymphangiogenesis induced by macrophages was promoted by IL-10, which is known to be expressed on CD11b macrophages^13,37^ and transform macrophages to an anti-inflammatory phenotype^38^. Based on the above findings, we speculate that anti-inflammatory macrophages may contribute to the enhancement of corneal lymphangiogenesis in bacterial keratitis, and that corneal lymphangiogenesis in bacterial keratitis affects one of the anti-inflammatory responses induced by macrophages.

We next focused on the role of corneal lymphangiogenesis in bacterial keratitis. We first confirmed that the bacterial load had no involvement in the late stage of bacterial keratitis. MacClellan *et al*.^31^ clarified that macrophages regulated inflammatory response caused by neutrophils, bacterial activity and cytokines in the early stage of *Pseudomonas aeruginosa*-induced keratitis. These findings suggest that corneal tissues are injured mainly by persistent inflammation via immune responses, rather than bacterial activity in the late stage of bacterial keratitis. Subsequently, our results showed that lymphangiogenesis improved corneal edema in the late stage of bacterial keratitis. In the clinical course of bacterial keratitis, corneal lymphangiogenesis may prevent aggravation of keratitis in the late stage of bacterial keratitis. Furthermore, our results indicated that corneal lymphangiogenesis contributed to the reduction of corneal opacity resulting from edema and inflammation. Previous studies have reported that corneal lymphangiogenesis aggravates herpetic keratitis^18^, dry eye^19^, and allergic disease^22^, and decreased corneal transplant survival rate^20^, suggesting unfavorable effects of corneal lymphangiogenesis. In contrast, Hos *et al*.^23,33^ suggested that lymphatic vessels supports the egress of macrophages from the cornea and contribute to reduce immune cells and acute edema, which are consistent with our results. Therefore, the present study indicates that corneal lymphangiogenesis via macrophages leads to resolution of bacterial keratitis, suggesting that induction of corneal lymphangiogenesis may be a novel treatment option of bacterial keratitis.

Our study has some limitations. First, we used clodronate liposomes to inhibit lymphangiogenesis via macrophages. Clodronate liposomes affect immune responses other than corneal lymphangiogenesis in bacterial keratitis, as described previously^31^. Hence we propose that corneal lymphangiogenesis plays an auxiliary role in the resolution of bacterial keratitis. Second, whether our results reflect a specific phenomenon in *Pseudomonas aeruginosa*-induced keratitis is unknown. The roles of corneal lymphangiogenesis remains controversial, and it is possible that corneal lymphangiogenesis plays different roles in keratitis caused by other etiologies such as Gram positive bacteria. We need to further study the roles of corneal lymphangiogenesis in keratitis models using other bacterial strains.

In summary, we studied the mechanism of corneal lymphangiogenesis related to macrophages in a murine bacterial keratitis model using *Pseudomonas aeruginosa*, and our findings showed that corneal lymphangiogenesis ameliorated corneal opacity and clinical keratitis score in the late stage, suggesting that corneal lymphangiogenesis may play a beneficial role by preventing the delay of wound healing in the late stage. Induction of corneal lymphangiogenesis may be a novel target for investigation of therapeutic approach to reduce corneal opacity induced by bacterial keratitis.

## Methods

### Animals and anesthesia

Female 8 to 10 week-old C57/BL6 mice were obtained from Japan CLEA (Shizuoka, Japan). All mice were housed in an animal facility at Tokyo Medical University. Experiments using mice were conducted according to the ARVO Statement for the Use of Animals in Ophthalmic and Vision Research. The study was approved by the Tokyo Medical University Institutional Animal Care and Use Committee. Animals were anesthetized by intraperitoneal injection of xylazine (5 mg/kg) and pentobarbitone (20 mg/kg) before any surgery.

### Bacterial keratitis model

We used the established mouse bacterial keratitis model by inoculating *Pseudomonas aeruginosa* (strain PAO-1) as described previously^34^. Strain PAO-1 was cultured on heart infusion agar for 24 hours at 35 °C and adjusted to a density of 1.0 × 10^8^ CFU/mL. Then, 2.5 μL of the suspension containing 2.5 × 10^5^ CFU was inoculated onto the cornea after epithelial injury was induced by scratching with a 27 G needle in the infected group. PBS was applied after epithelial scratching in the control group.

### Immunostaining and image analyzing

We evaluated lymphangiogenesis, angiogenesis, and macrophage infiltration by immunostaining of whole-mount corneas on days 2, 7 and 14 post-inoculation. Whole mount corneas were fixed in 99.5% ethanol for 20 min at 4 °C. After rinsing three times in PBS, samples were incubated with 5% bovine serum albumin, 5% goat serum and 0.3% Triton X-100 for 1 h at room temperature to block nonspecific reaction. Primary antibody (1:100) with 1% bovine serum albumin and 0.1% Triton X-100 was applied to the corneas overnight at 4 °C. The following primary antibodies were used: anti-mouse CD31 antibody (PE-conjugated, BioLegend, San Diego, CA, USA), anti-mouse/human CD11b antibody (PE-conjugated, BioLegend), anti-mouse F4/80 antibody (PE-conjugated, TONBO Biosciences, San Diego, CA, USA) and anti-rabbit LYVE-1 antibody (AngioBio, Del Mar, CA, USA). The corneas were rinsed three times in PBS containing 0.1% Nonidet P-40. For LYVE-1 immunostaining, Alexafluor488–conjugated anti-goat-rabbit (1:1000; Life technologies, Eugene, OR, USA) was used as the secondary antibody, and was applied with 1% bovine serum albumin and 0.1% Triton X-100 for 1 h at room temperature. The immunostained corneas were flattened and photographed under a fluorescence microscope (Olympus BX51 and DP70; Olympus Corporation, Tokyo, Japan) and a confocal microscope (LMS700, Carl Zeiss, Thornwood, NY, USA). Image J software (National Institute of Health, Bethesda, Maryland, USA) was used to calculate the percentage of vessel-covered area, as described previously^21^. The perforated corneas were excluded from analysis.

### RT and Quantitative Real-Time PCR

We harvested corneas from mice with bacterial keratitis on days 2 and 9 post-inoculation as the time of start of angiogenesis and lymphangiogenesis, respectively. Corneas inoculated with PBS after scratching on day 2 post-inoculation were used as controls. Total RNA was isolated using miRNeasy Mini Kit (Qiagen, Valencia, CA, USA), and reverse-transcribed using Transcript First Stand cDNA Synthesis Kit (Roche, Basel, Switzerland). Real-time PCR was performed using TaqMan Universal PCR Mastermix (Applied Biosystems, Foster City, CA, USA) and preformulated primers for VEGF-A (NM_001025250.3), VEGF-C (NM_009506.2) and VEGFR-3 (NM_008029.3), and the Light Cycler 96 System (Roche, Basel, Switzerland). The results were analyzed by the comparative threshold cycle method and normalized by GAPDH as internal control.

### Depletion of corneal macrophages

Mice were given intraperitoneally injection of 200 μL of clodronate-containing liposomes (7 mg/mL, Formumax Scientific, Sunnyvale, CA, USA) as described previously^39^. In clodronate (+) group, the clodronate liposomes were injected at the same time as bacterial inoculation. The clodronate liposomes were injected on days −2 (day 2 pre-inoculation), 2, and 6 post-inoculation in the early macrophage (−) group, or on days 4, 8 and 12 post-inoculation in the late macrophage (−) group. Mice that did not receive clodronate liposome injection were used as controls [macrophage (+) group].

### Bacterial load

The bacterial load was measured as described previously^31^. The corneas were harvested and homogenized on days 2, 7 and 14 post-inoculation. These samples were plated on *Pseudomonas aeruginosa* isolation agar, and the numbers of colonies on the plates were counted. The results are expressed as log_10_ CFU/cornea.

### Evaluation of corneal edema

Corneal edema was evaluated by measuring central corneal thickness (CCT) using AS-OCT (CASIA SS-1000; Tomey, Nagoya, Japan). The OCT system achieves high resolution imaging of 10 µm (axial) and 30 µm (transverse) and scanning of 30,000 A-scans per second. Corneas were scanned and analyzed using the OCT software. CCT was calculated by measuring the distance between the top and the bottom of corneal endothelium. These measurements were performed in duplicate in each eye by the same investigator who was masked to the group. Perforated corneas were excluded from analysis.

### Clinical infection scoring

This grading procedure has been described previously^31^: 0; clear or slight opacity, partially or fully covering the pupil, 1; slight opacity, fully covering the anterior segment, 2; dense opacity, partially or fully covering the pupil, 3; dense opacity, covering the entire anterior segment, and 4; corneal perforation or phthisis.

### Statistical analysis

The significance of differences between means was determined using Student t-test. Error bars in figures were expressed as mean ± SD, and p values less than 0.05 were considered statistically significant. All analyses were performed using Microsoft Excel (Microsoft Corp, Redmond, WA, USA) and SPSS (SPSS ver. 22.0; SPSS Inc., Chicago, IL, USA) software.

## Supplementary information

Supplementary Dataset
