# Supplementary material for: Corneal lymphangiogenesis ameliorates corneal inflammation and edema in late stage of bacterial keratitis
Source: Sci Rep. 2019 Feb 27;9:2984. doi: 10.1038/s41598-019-39876-x (PMC6393676; doi:10.1038/s41598-019-39876-x)
Supplement: Supplementary file 1 — Supplementary Dataset [file 41598_2019_39876_MOESM1_ESM.pdf]

Corneal lymphangiogenesis ameliorates corneal inflammation and edema in late stage of bacterial keratitis

Akitomo Narimatsu[1, 2], Takaaki Hattori[1*], Naohito Koike[2], Kazuki Tajima[3], Hayate Nakagawa[1], Naoyuki Yamakawa[1], Yoshihiko Usui[1], Shigeto Kumakura[1], Tetsuya Matsumoto[1, 4] and Hiroshi Goto[1]

[1] Department of Ophthalmology, Tokyo Medical University, Tokyo, Japan
[2] Department of Microbiology, Tokyo Medical University, Tokyo, Japan
[3] Department of Small Animal Internal Medicine, School of Veterinary Medicine, University of Kitasato, Aomori, Japan
[4] Department of Infectious Diseases, International University of Health and Welfare, Narita, Japan

Correspondence and requests for materials should be addressed to Takaaki Hattori (email: ninnin@ss.iij4u.or.jp)

Supplemental figures:

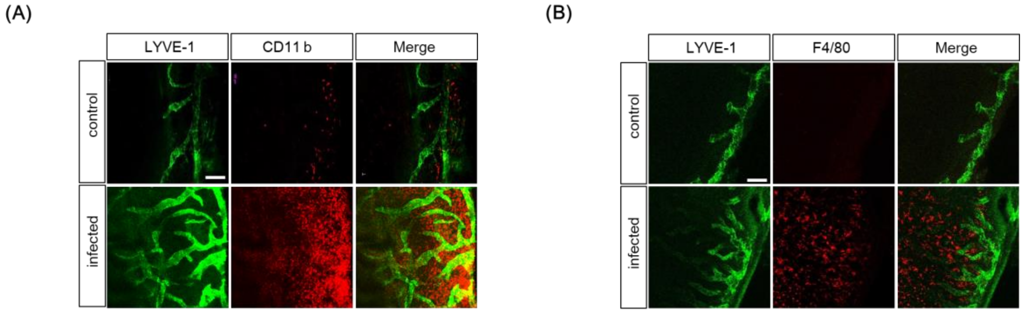

Supplementary Fig. S1. Co-localization of CD11b positive, F4/80 positive macrophages and corneal lymphatic vessels in the late stage of bacterial keratitis. **(A)** Corneas were immunostained by anti-CD11b antibody (red) and anti-LYVE-1 antibody (green). **(B)** Corneas were immunostained by anti-F4/80 antibody (red) and anti-LYVE-1 antibody (green). The images were captured by confocal microscopy on day 14 post-inoculation. Scale bar: 100 μm.

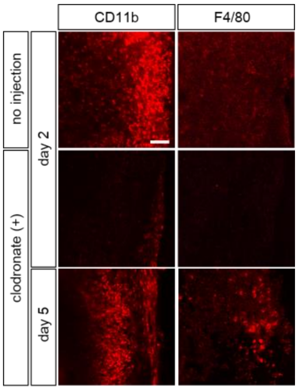

Supplementary Fig. S2. The effect of a single intraperitoneal injection of clodronate liposomes on the infiltration of CD11b positive macrophages and F4/80 positive macrophage into the cornea. In clodronate (+) group, the clodronate liposomes were injected at the same time as bacterial inoculation. Corneas were immunostained by anti-CD11b antibody and anti-F4/80 antibody on days 2 and 5 post-inoculation, and images were captured by confocal microscopy. Scale bar: 100μm.

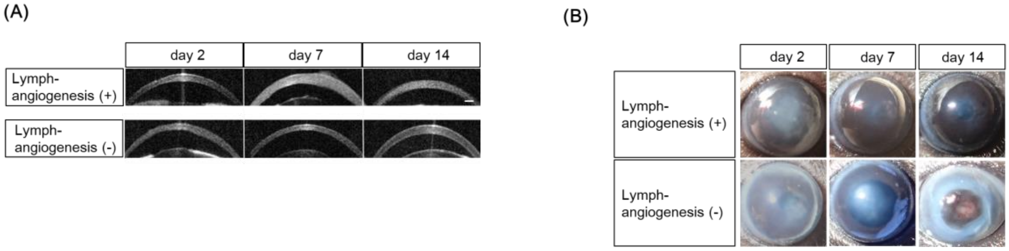

Supplementary Fig. S3. (A) Representative photographs of central corneal thickness measured by AS-OCT in lymphangiogenesis (+) group and lymphangiogenesis (-) group on day 14 post-inoculation. Scale bar: 200 μm. **(B)** Representative photographs of corneal opacity in lymphangiogenesis (+) group and lymphangiogenesis (-) group on day 14 post-inoculation.
